# Clinical characteristics, socioeconomic factors and COVID-19 were associated with delayed surgery in children with hypospadias: a retrospective study of 4439 cases in a single center

**DOI:** 10.1186/s40001-022-00744-6

**Published:** 2022-07-18

**Authors:** Gaochen Bai, Feng Liang, Tianxin Zhao, Fuming Deng, Kai Fu, Xiong Chen, Zhongmin Li, Liyu Zhang, Wei Jia, Wen Fu, Guochang Liu

**Affiliations:** 1 Department of Pediatric Urology, Guangzhou Women and Children’s Medical Center, Guangzhou Medical University, No.9 Jinsui Road, 510623 Guangzhou, China; 2 Department of Pediatric Surgery, Guangzhou Institute of Pediatrics, Guangzhou Women and Children’s Medical Center, Guangzhou Medical University, No.9 Jinsui Road, 510623 Guangzhou, China; 3 Clinical Data Center, Guangzhou Women and Children’s Medical Center, Guangzhou Medical University, No.9 Jinsui Road, 510623 Guangzhou, China

**Keywords:** Hypospadias, Delayed surgery, Clinical characteristics, Socioeconomic factors, COVID-19

## Abstract

**Background:**

Hypospadias is one of the most common congenital diseases of the genitourinary system in children. The European Association of Urology (EAU) Guidelines recommend that children undergoing hypospadias surgery should be between 6 and 18 months. In China, where many children have hypospadias, it remains unknown whether clinical characteristics, socioeconomic factors and COVID-19 were associated with delayed surgery in children with hypospadias.

**Methods:**

We retrospectively analyzed children with hypospadias who underwent primary surgery at the Department of Pediatric Urology in Guangzhou Women and Children’s Medical Center between January 2010 and October 2021. Patients who had two-stage surgery or a second round of surgery due to complications were excluded to eliminate data duplication. The clinical characteristics and demographic information were collected. We defined delayed surgery as primary surgery performed after 18 months following the EAU Guidelines.

**Results:**

A total of 4439 children diagnosed with hypospadias were included in the study. The median age (29.1 ± 16.7 months) of surgery for hypospadias in our study was much higher than the recommended age reported in the EAU guidelines, and 76.6% of the children underwent surgery after the age of 18 months. Children without comorbidities including cryptorchidism (odds ratio [OR] = 1.562; 95% confidence interval [CI] 1.199–2.034; *p* = 0.001), prostatic cyst (OR = 2.613; 95% CI 1.579–4.324; *p* < 0.001), penile hypoplasia (OR = 1.778; 95% CI 1.225–2.580; *p* = 0.002), inguinal hernia (OR = 2.070; 95% CI 1.394–3.075; *p* < 0.001), and penoscrotal transposition (OR = 4.125; 95% CI 1.250–13.619; *p* = 0.020) were more likely to receive delayed surgery. Living in a low economic area (OR = 1.731; 95% CI 1.068–2.806; *p* = 0.026) or not close to a main medical center (OR = 1.580; 95% CI 1.370–1.824; *p* < 0.001) was highly associated with delayed surgery. The proportion of children undergoing delayed surgery and the median age of surgery during the COVID-19 pandemic were significantly higher than those before the COVID-19 pandemic (*p* = 0.004 and < 0.001, respectively).

**Conclusions:**

Most children with hypospadias received delayed surgery (surgical age > 18 months). Comorbidities, living in a low economic area, too far from a main medical center and the COVID-19 pandemic were highly associated with delayed surgery. It is vital to improve the public awareness of hypospadias and strengthen the re-education of primary community doctors to reduce delayed surgery.

## Introduction

Hypospadias is one of the most common congenital diseases of the genitourinary system in children, with an incidence rate of 1/150–1/250. The incidence has recently increased in many countries and regions [1, 2]. Its main characteristic is ectopic urethral opening, and certain patients are accompanied by chordee, which seriously affects physical and mental health.

Surgery is currently the only effective treatment for hypospadias, including primarily penile straightening, urethroplasty, and appearance shaping [3]. The timing of corrective surgery to treat hypospadias has always been controversial, with the widespread community and medical concerns. Some studies have found that increasing surgical age can lead to poor surgical outcomes and a significant increase in complications [4, 5]. Additionally, when surgery is undertaken, the patient’s age has long-term implications for affecting sexual behavior and development, both important considerations when deciding treatment. Most children develop gender consciousness from around 18 months, then gradually establish urination habits. Children’s memory of early surgery is conducive to their mental health development after adolescence, and early surgery can avoid challenges caused by urination problems and improve their quality of life and social adaptability [6, 7]. In 2021, the European Association of Urology (EAU) Guidelines on Pediatric Urology recommended that children undergoing hypospadias surgery should be between 6 and 18 months [8]. However, there are still many children with hypospadias whose surgical age is far beyond the recommended age. A study of 89 patients in Taiwan, China, showed that the factors leading to delayed hypospadias surgery in children included the age and specialty of physicians at the first visit, residential urbanization level, and the presence of concomitant cryptorchidism, prematurity or low birth weight [9]. However, no large-scale or long-period studies have confirmed these reported outcomes.

Since the COVID-19 infection outbreak at the end of 2019 and its global spread, medical resources have been depleted, with medical decision-making undergoing tremendous changes. Most medical and surgical societies have developed recommendations and triage systems to guide decision‑making, focusing on adult patients or pediatric surgery [10, 11]. As Wei et al. [12] reported in China, during the COVID-19 pandemic, 62.86% fewer patients visited the hospital and underwent pediatric operations relative to the corresponding period one year earlier (*p* < 0.01). After hospitals and clinics made protocols and reorganized healthcare facilities in response, the number of operations performed remained stable and comparable to the pre-pandemic period. The COVID-19 pandemic has also reduced population movements, making it more inconvenient for patients to visit hospitals. These conditions may impede the timely consultation and treatment of children with hypospadias.

In China, where many children have hypospadias, identifying the factors that cause delays in surgery can help guide medical decision-making and improve the physical and mental health outcomes for children with hypospadias and the comprehensive management effectiveness of hypospadias, especially during the COVID-19 pandemic.

## Methods

### Study design and patients

**Fig. 1:**
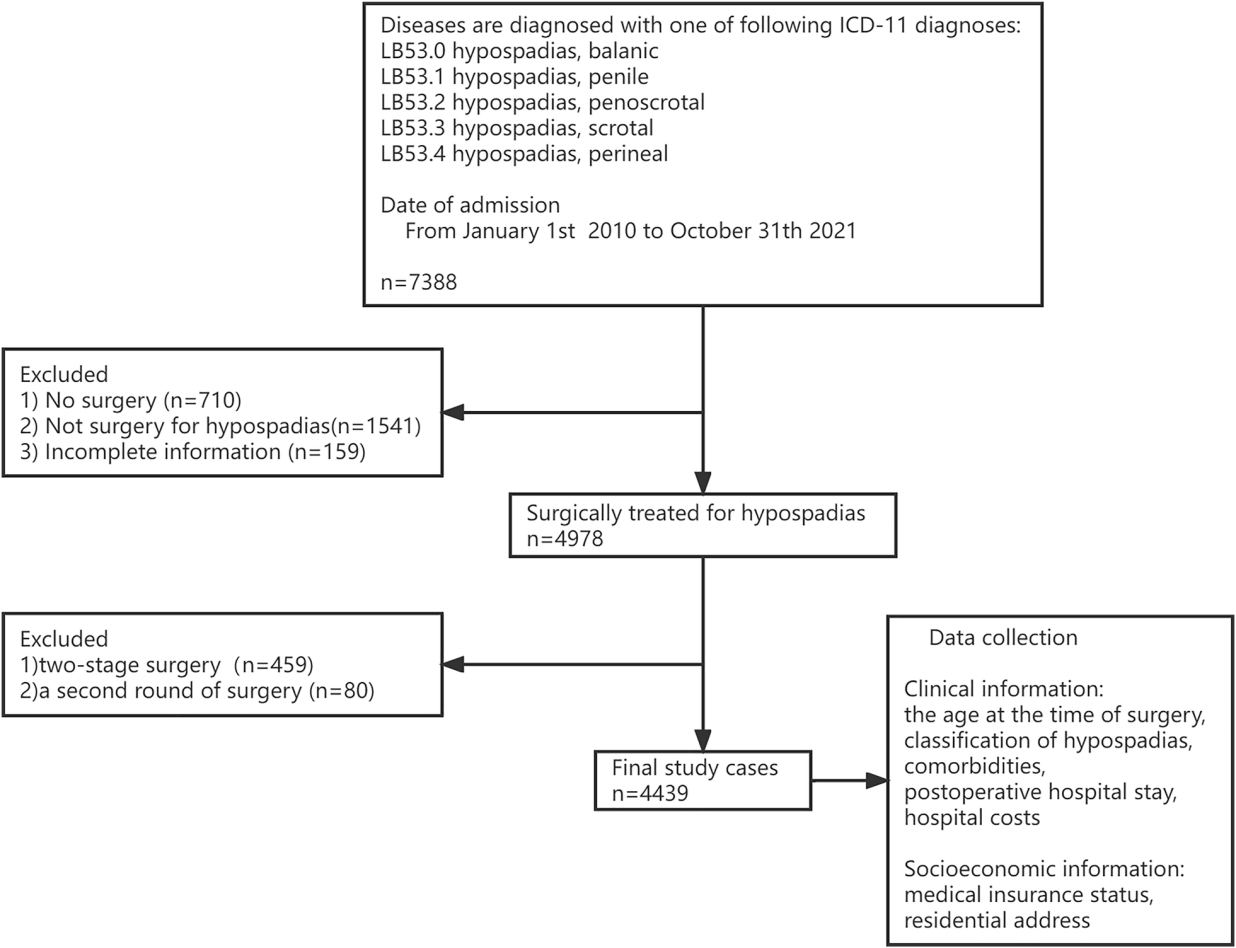
Flowchart of the retrospective protocol

We retrospectively analyzed children with hypospadias who underwent primary surgery at the Department of Pediatric Urology in Guangzhou Women and Children’s Medical Center between January 2010 and October 2021 (Fig. 1). The study was approved by the ethics committees of Guangzhou Women and Children’s Medical Center. The need for informed consent was waived because of the study’s retrospective nature. Patients who had two-stage surgery or a second round of surgery due to complications were excluded to eliminate data duplication. The clinical characteristics and demographic information were collected, including the age at the time of surgery, classification of hypospadias, comorbidities, postoperative hospital stay, hospital costs, and socioeconomic factors including medical insurance status and residential address. The classification of hypospadias was according to the EAU Guidelines. Regional economic data were determined using the per capita Gross Domestic Product (GDP) and low economic areas were defined as per capita GDP < 5000 United States dollars (USD). People living in Guangzhou or the seven cities adjacent to Guangzhou were considered as neighboring a main medical center.

### Definition of delayed surgery

The EAU Guidelines recommend that surgical treatment of hypospadias should be performed between 6 and 18 months [8], and we defined delayed surgery as primary surgery performed after 18 months.

### Statistical analyses

Statistical analysis was performed using GraphPad Prism Software 8.3 (La Jolla, California, USA). Categorical variables were presented as ratios (%), and continuous variables were presented by mean ± standard deviation (SD) and median (interquartile range). Comparisons were undertaken using a χ2 test or Fisher’s exact test for dichotomous outcomes. Normally and non-normally distributed continuous variables were compared using Student’s *t*-test and Mann–Whitney *U* test. Variables that were determined to be significant in the univariate analysis of timely and delayed surgery of hypospadias (6–18 months compared to > 18 months) were included in the logistic regression analysis. A *p*-value less than 0.05 was considered statistically significant.

## Results

From the pool of potential candidates over the 11 years of the study, a total of 4439 children diagnosed with hypospadias were included (Table 1). The median age (29.1 ± 16.7 months) of surgery for hypospadias in our study was much higher than the recommended age reported in the EAU guidelines, and 76.6% (3402/4439) of the children underwent surgery after the age of 18 months. The proportion of proximal hypospadias was 45.2%, much higher than reported in other countries and regions [13, 14]. The most common comorbidities were hydrocele (7.7%), cryptorchidism (6.6%), and prostatic cyst (3.8%). The average postoperative hospital stay was 7.13 days, and the median hospital cost was approximately 17,366 yuan (2726 USD). 3.4% of the children lived in low economic areas, but the majority (71.4%) had medical insurance to cover most of the hospital costs.

**Table 1:** Demographic, clinical, and socioeconomic characteristics of 4439 cases

Characteristic	Total (*n* = 4439)	Timely surgery (*n* = 1037)	Delayed surgery (*n* = 3402)	*p-*value
Age at surgery (month), median (IQR)	27 (19, 34)	15 (13, 17)	30 (24, 36)	< 0.001
Average age (month), mean ± SE	29.1 ± 16.7	14.7 ± 2.5	33.4 ± 16.7	< 0.001
Classification, *n* (%)				
Distal-anterior	336 (7.6)	66 (6.4)	270 (7.9)	< 0.001
Intermediate-middle	2095 (47.2)	560 (54.0)	1535 (45.1)^a^	
Proximal-posterior	2008 (45.2)	411 (39.6)	1597 (46.9)	
Comorbidity, *n* (%)				
Cryptorchidism	295 (6.6)	92 (8.9)	203 (6.0)	0.001
Hydrocele	340 (7.7)	81 (7.8)	259 (7.6)	0.834
Prostatic cyst	167 (3.8)	18 (1.7)	149 (4.4)	< 0.001
Penile dysplasia	133 (3.0)	47 (4.5)	86 (2.5)	0.001
Inguinal hernia	111 (2.5)	43 (4.1)	68 (2.0)	< 0.001
Chordee	80 (1.8)	15 (1.4)	65 (1.9)	0.325
Penoscrotal transposition	47 (1.1)	19 (1.8)	28 (0.8)	0.005
Other genital anomalies	157 (3.5)	38 (3.7)	119 (3.5)	0.799
Urinary system diseases	41 (0.9)	3 (0.3)	38 (1.1)	0.015
Medical insurance, *n* (%)	3171 (71.4)	720 (69.4)	2451 (72.0)	0.103
Poverty county residence, *n* (%)	152 (3.4)	20 (1.9)	132 (3.9)	0.002
Neighboring a main medical center, *n* (%)	2126 (47.9)	589 (56.8)	1537 (45.2)	< 0.001
Postoperative hospital stay (day), mean ± SE^b^	7.13 ± 2.123	6.79 ± 1.987	7.24 ± 2.152	< 0.001
Hospital costs (CNY/USD), median (IQR)	17,366.0 (14,650.3, 21,431.2)	18,258.2 (16,037.7, 20,534.5)	16,888.2 (16,142.2, 21,765.7)	< 0.001

^a^Compared to distal-anterior group and proximal-posterior group, both of *p*-values < 0.017; *CNY* Chinese yuan; *USD* United States dollar

^b^Defined as time from surgery to discharge

**Fig. 2:**
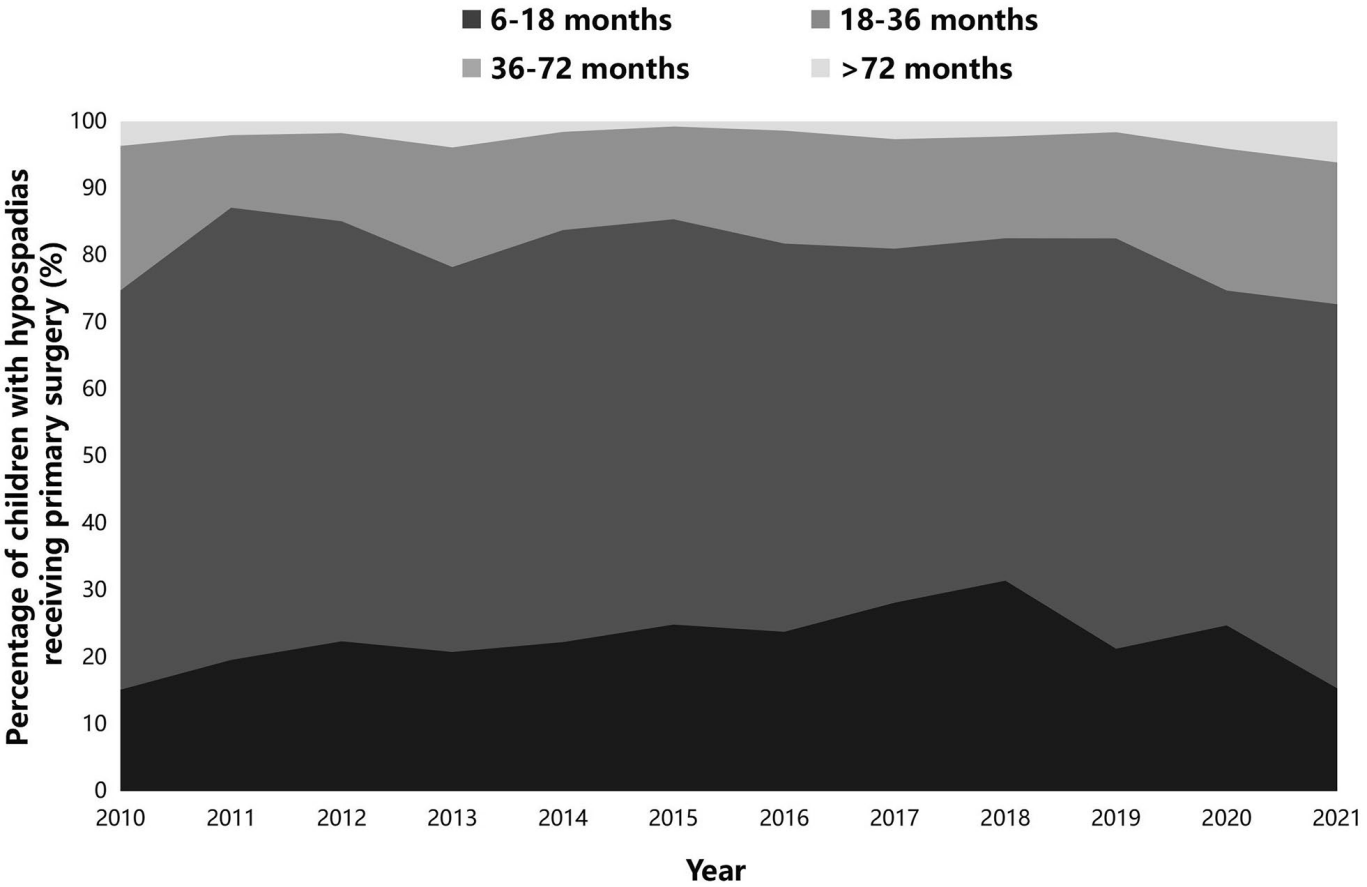
Percentages of children with hypospadias receiving primary surgery from 2010 to 2021 by age category

The number of children who had timely surgery rose from 15.1% in 2010 to 31.4% in 2018 but dropped to 15.3% in 2021 (Fig. 2). Among children with delayed surgery, the proportion of cryptorchidism, prostatic cyst, penile dysplasia, inguinal hernia, penoscrotal transposition, and urinary system diseases was lower than that among children with timely surgery (all of the *p*-values < 0.05). There were no differences in the proportion of hydrocele, chordee, and other genital anomalies between the children with timely and delayed surgery (Table 1).

Children living in low economic areas and not close to a main medical center were more likely to receive delayed surgery (Table 1). However, the medical insurance status of the two groups was similar. The delayed surgery group’s postoperative hospital stay and hospital costs were lower than those in the timely surgery group, probably because younger children with poor physical development and weak immunity were more prone to postoperative complications, such as pneumonia, enteritis, and urinary tract infections.

Logistic regression analysis in Table 2 shows that children without comorbidities including cryptorchidism (odds ratio [OR] = 1.562; 95% confidence interval [CI] 1.199–2.034; *p* = 0.001), prostatic cyst (OR = 2.613; 95% CI 1.579–4.324; *p* < 0.001), penile hypoplasia (OR = 1.778; 95% CI 1.225–2.580; *p* = 0.002), inguinal hernia (OR = 2.070; 95% CI 1.394–3.075; *p* < 0.001), and penoscrotal transposition (OR = 4.125; 95% CI 1.250–13.619; *p* = 0.020) were more likely to receive delayed surgery. Living in a low economic area (OR = 1.731; 95% CI 1.068–2.806; *p* = 0.026) or not close to a main medical center (OR = 1.580; 95% CI 1.370–1.824; *p* < 0.001) was highly associated with delayed surgery.

**Table 2:** Logistic regression analysis of factors associated with delayed surgery

Factors	Parameter estimate	SE	Chi-squared test	*p*-value	OR (95% CI)
No cryptorchidism	0.446	0.135	10.960	0.001	1.562 (1.199–2.034)
No prostatic cyst	0.961	0.257	13.978	< 0.001	2.613 (1.579–4.324)
No penile dysplasia	0.575	0.190	9.186	0.002	1.778 (1.225–2.580)
No inguinal hernia	0.728	0.202	12.996	< 0.001	2.070 (1.394–3.075)
No penoscrotal transposition	0.880	0.305	8.328	0.004	2.411 (1.326–4.382)
No urinary system diseases	1.417	0.609	5.408	0.020	4.125 (1.250–13.619)
Living in poverty county	0.549	0.246	4.960	0.026	1.731 (1.068–2.806)
Not neighboring a main medical center	0.458	0.073	39.253	< 0.001	1.580 (1.370–1.824)

*SE* standard error; *OR* odds ratio; *CI* confidence interval

**Fig. 3:**
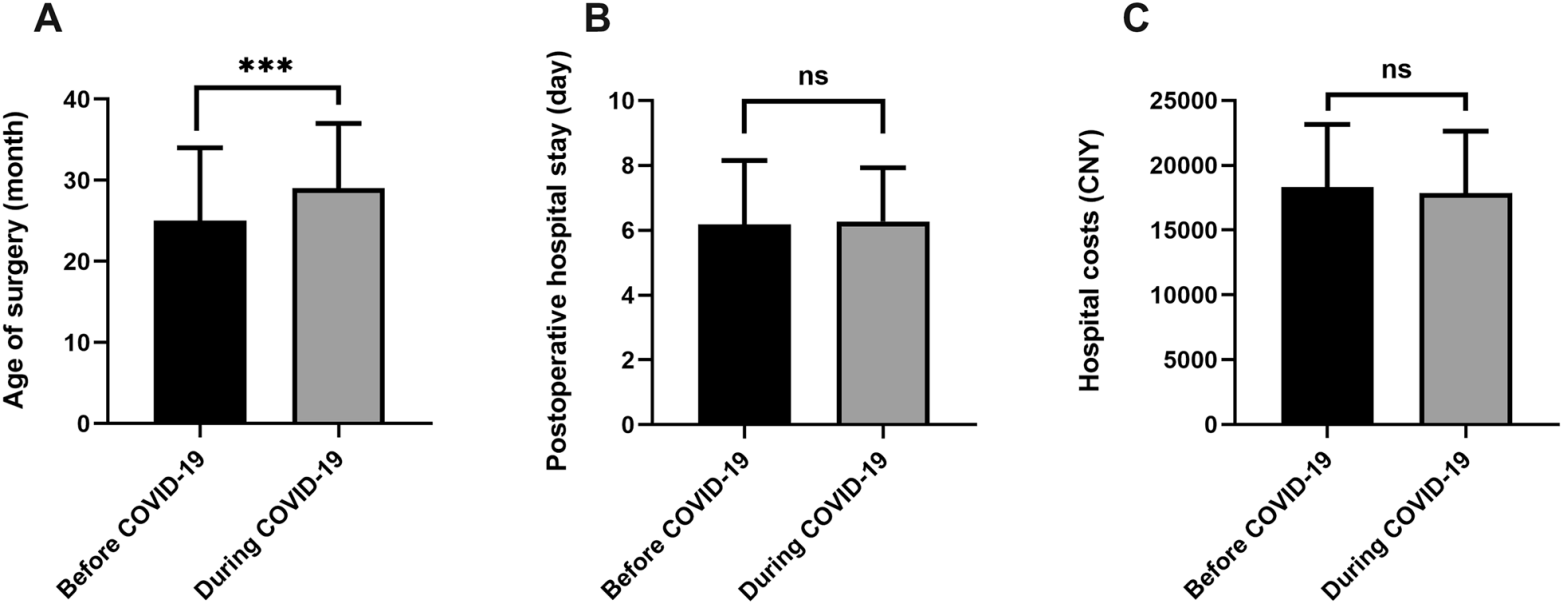
Comparison of children with hypospadias receiving primary surgery before and during the COVID-19 pandemic. ***p < 0.001

Finally, January 1, 2020 was determined to be the cutoff point to compare children undergoing hypospadias surgery before and during the COVID-19 pandemic (2018–2019 vs. 2020–2021) (Fig. 3). The proportion of children undergoing delayed surgery during the COVID-19 pandemic (79.9%, 556/696) was higher than that before the COVID-19 pandemic (73.9%, 786/1064), and the difference was statistically significant (*p* = 0.004). The median age of surgery during the COVID-19 pandemic was significantly higher than that before the COVID-19 pandemic (*p* < 0.001), even though no difference in postoperative hospital stay and hospital costs in these two periods was observed.

## Discussion

Although the exact timing of surgery when treating children with hypospadias is still controversial, it is widely accepted that patients should undergo early surgical repair [8, 15–17]. EAU guidelines recommend surgery performed between 6 to 18 months as mentioned above, however, the mean age of surgery in our study was 29.1 months and only 23.4% of children underwent surgery before 18 months of age. An overview published in 2012 summarizing 113 clinical studies of hypospadias corrective surgeries over the past decade found significant differences in the age of primary surgery of hypospadias in North America, Europe, and China. The average age of 20.6 months, 31.7 months, and 67.1 months, respectively, in these three large areas [18]. A recent multicenter study involving 1386 patients in China reported that the surgical age for hypospadias ranged from 6 to 180 months, with a median of 29 months [19]. It is pleasing to see that the average age of primary surgery in our study was younger than that in previous studies.

Specific clinical characteristics were associated with delayed surgery for children with hypospadias, including prostatic cyst, penile hypoplasia, oblique inguinal hernia, penoscrotal transposition, and urinary system diseases. Snodgrass et al. [20] reported that 86% of parents had never heard of hypospadias when told of their newborn diagnosis. It is apparent that parents were more likely to take their children to the hospital when multiple anomalies were easily spotted in the perineum. These results suggest an urgent need to raise public awareness for hypospadias to enable earlier prevention, diagnosis, and treatment. The outcome of our study inferred that hypospadias classification and chordee would not affect the surgical age, which was different from a previous report in China [21]. This may be because parents are very concerned about children's genitals, and they will take their children to hospitals for any slight anomalies.

Regarding socioeconomics, children living in low economic areas and not close to a medical center had a higher risk of delayed surgery, consistent with previous findings of delayed surgery for cryptorchidism and testicular torsion [22, 23]. Newborns with hypospadias are generally examined and given preliminary treatment advice by the primary community physicians. The specialized knowledge of primary care facilities is challenging to update. It may restrict the ability of such facilities to give an accurate diagnosis and appropriate treatment advice, especially in low economic areas. Although primary-level medical care is making significant progress in China, medical and health resources in low economic areas are relatively scarce, especially access to pediatricians and clinicians with pediatric expertise [24, 25]. Re-education and training for primary care facilities and rebalancing the allocation of health care resources may enable more children with hypospadias to have access to timely surgery, especially in low economic areas.

The spread of COVID-19 infection has posed many challenges to the supply and balance of medical resources and inconvenienced travel. By analyzing the surgical age of children with hypospadias before and during the COVID-19 pandemic, it was found that a higher proportion of children did not have timely surgery during the COVID-19 pandemic. Quaedackers et al. [26] developed recommendations for pediatric urology cases based on published research and expert opinions, suggesting that most children’s urinary diseases were not urgent and did not require immediate interventions. However, delayed treatment may negatively impact future fertility or kidney function in these children, and it is unclear how long the COVID-19 pandemic will last. Garnier et al. [27] investigated 501 boys who underwent surgery for hypospadias and found that surgical age > 2 years was a significant predictor of complications (*p* = 0.002, OR = 1.98). Dyssynergia was more common between the ages of 24 and 36 months (12.5% vs. 3.6%; *p* = 0.01) and healing problems were more common in boys aged > 13 years (1.5% vs. 28.5%; *p* = 0.06). Early hypospadias repair before 18 months of age can minimize the potential psychological trauma caused by surgery, such as rapprochement, stranger anxiety, and separation anxiety, enabling better adaptation to social roles. Pediatric urologists need to redress the delayed surgery caused by COVID-19 and adopt new countermeasures, such as online clinics and increased day surgery rates [28, 29]. Liu et al. [30] reported that online psychological counseling services and social software had been widely used during the COVID-19 pandemic, which could enable health authorities to allocate health resources and develop appropriate treatments for medical staff and the public who had mental health problems. These successful experiences can be applied to pediatric urology to increase the convenience of patients. Finally, strengthening parental education and readjusting the priorities of pediatric urogenital surgery according to patients’ age, clinical characteristics, and socioeconomic factors may help more children with hypospadias to receive timely diagnosis and treatment.

This study has several limitations. Firstly, the outcome is limited by the nature of retrospective observations. More detailed clinical and socioeconomic information that might be relevant to delayed surgery was not available, such as parental education level, parental awareness of hypospadias, the presence of siblings, and family income. Secondly, because the data were from a single pediatric institution and the economic development of Guangzhou and its nearby areas is higher than the national average, some degree of selection bias may exist in this study. Thirdly, although it is common in clinical work that some children with hypospadias or their parents prefer delayed surgical intervention due to the concerns about the risk of anesthesia and unexpected surgical complications or voluntarily miss medical visits due to the COVID-19 fear and spread, the specific percentage of the study population is hard to draw conclusions from this retrospective study. Further prospective studies can include questionnaires or telephone follow-ups to accurately evaluate the prevalence and associated factors of surgery delays caused by such conditions. As over 4400 cases were included in the study, with all the data points being complete and reliable, the conclusions from this large-scale clinical study are appropriately representative of children in China.

## Conclusions

Approximately 76.6% of children with hypospadias in this study received delayed surgery (surgical age > 18 months). Children without comorbidities including cryptorchidism, prostatic cyst, penile hypoplasia, inguinal hernia, penoscrotal transposition, and urinary system diseases were more likely to receive delayed surgery. Living in a low economic area or too far from a major city medical center was also highly associated with delayed surgery. The COVID-19 pandemic has contributed to increasing surgical age and the proportion of delayed surgery in children with hypospadias. It is vital to improve the public awareness of hypospadias and strengthen the re-education of primary community doctors to reduce delayed surgery. The health department and pediatric urologists should adopt improved countermeasures to avoid the negative impacts of the COVID-19 pandemic on such time-sensitive treatment as that required for patients with hypospadias.

## Data Availability

The datasets used and/or analyzed during the current study are available from the corresponding author on reasonable request.

## Abbreviations

EAU: European Association of Urology
GDP: Gross domestic product
CNY: Chinese yuan
USD: United States dollars
SD: Standard deviation
SE: Standard error
OR: Odds ratio
CI: Confidence interval
